# Dynamic Modeling of Reversible Methanolysis of *Jatropha curcas* Oil to Biodiesel

**DOI:** 10.1155/2013/268385

**Published:** 2013-12-02

**Authors:** Azhari M. Syam, Hamidah A. Hamid, Robiah Yunus, Umer Rashid

**Affiliations:** ^1^Institute of Advanced Technology, Universiti Putra Malaysia (UPM), 43400 Serdang, Selangor, Malaysia; ^2^Department of Chemical Engineering, Malikussaleh University, Lhokseumawe 24351, Indonesia

## Abstract

Many kinetics studies on methanolysis assumed the reactions to be irreversible. The aim of the present work was to study the dynamic modeling of reversible methanolysis of *Jatropha curcas* oil (JCO) to biodiesel. The experimental data were collected under the optimal reaction conditions: molar ratio of methanol to JCO at 6 : 1, reaction temperature of 60°C, 60 min of reaction time, and 1% w/w of catalyst concentration. The dynamic modeling involved the derivation of differential equations for rates of three stepwise reactions. The simulation study was then performed on the resulting equations using MATLAB. The newly developed reversible models were fitted with various rate constants and compared with the experimental data for fitting purposes. In addition, analysis of variance was done statistically to evaluate the adequacy and quality of model parameters. The kinetics study revealed that the reverse reactions were significantly slower than forward reactions. The activation energies ranged from 6.5 to 44.4 KJ mol^−^1.

## 1. Introduction

Methanolysis is the most common and cheapest biodiesel production route. In general, this process may be carried out simultaneously by employing either homogeneous catalysts such as alkalis [1–3], acids [4, 5], and enzymes [6, 7] or heterogeneous catalysts [8–10]. Borges and Díaz [11] have also performed the reaction using heterogeneous catalyst. The homogeneous alkali catalysts often suffer problems such as difficulty in removing the catalyst after the reaction process, utilization of large amount of water for purification, and emulsification as well [12].

**Figure 1:**
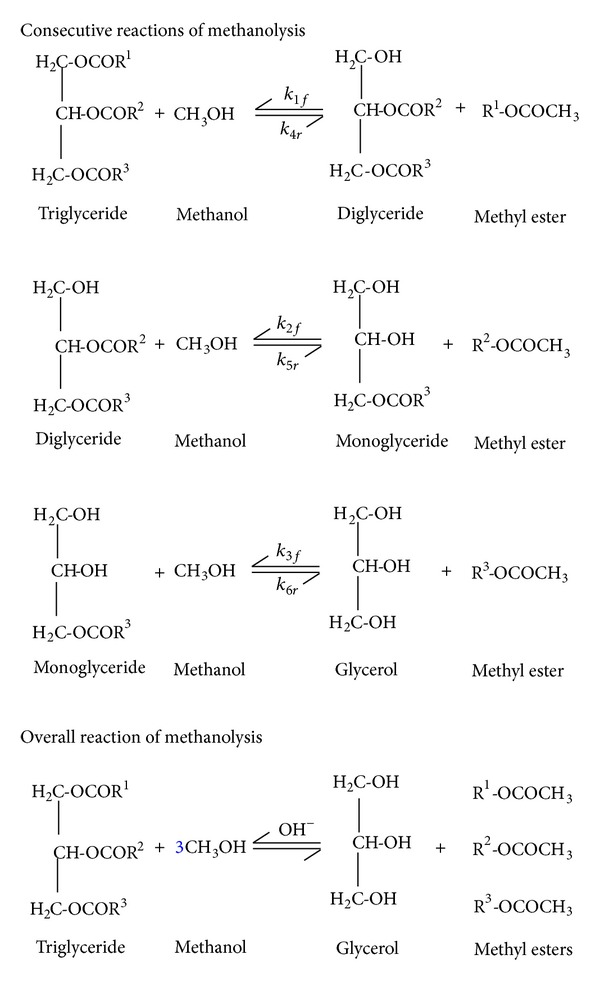
Mechanisms of consecutive and overall reaction of methanolysis.

Theoretically, the methanolysis process takes place under three consecutive reactions as shown in Figure 1. Firstly, triglyceride molecule reacts with methanol to produce methyl ester and intermediate molecule of diglyceride. Then, diglyceride reacts again with methanol and produces intermediate molecule of monoglyceride and methyl ester. Finally, monoglyceride reacts with methanol to produce the final product, glycerol and methyl ester. Thus, based on the stoichiometric equation, the overall reaction requires one mole of triglyceride molecule to react with three moles of methanol to produce one mole of glycerol and three moles of methyl esters. Cheng et al. [13] reported that the reactions were mainly endothermic in nature.

The methanolysis reaction takes place very fast, in particular, when the excess methanol is used to avoid the backward reaction. If the backward reaction happens, the formation of product will take a longer time to complete because the reaction will never reach an equilibrium condition. For these reasons stepwise reactions cannot go on under a normal reaction mechanism. Sometimes the formation of intermediate products can be detected by the analytical analysis such as gas chromatography. However, in most cases the intermediate compound cannot be identified at all because of the nature of simultaneous reactions. Therefore, most of the kinetics studies on methanolysis reaction assumed that the stepwise reactions proceed in an irreversible mode [14–17], thus requiring very simple modeling approach. Similar studies on modeling biodiesel have been done also by other researchers [18–20], those of which focused particularly on the kinetics of homogeneous alkali catalyzed methanolysis in batch reactor. Thus, the existing kinetics models are only applicable under certain prescribed conditions that support irreversible assumption. However with the advent of technology in numerical computation, visualization, and programming, this assumption is no longer necessary. Using MATLAB, the complicated kinetics equations for the reversible reactions can be solved using multiple approaches and can reach a solution faster. Using these models both forward and backward reaction rates, an Arrhenius activation energy can be determined at every reaction temperature.

This paper focuses on modeling of methanolysis process using *Jatropha curcas* oil (JCO) as feedstock. Most researchers had developed some models on biodiesel production as well as biodiesel applications; however, literatures on modeling of biodiesel synthesis using JCO are still limited. Another study related to modeling of biodiesel production process, in particular about the separation method of by-product (glycerol) to obtain a standardized biodiesel quality, has been developed [21]. In terms of reactor configuration, the system has been modeled and simulated by Cheng et al. [13] who used a membrane reactor and prereactor. Their findings reported that the prereactor may be needed as the initial stage for carrying out a substantial part of the methanolysis process. Thus, the subsequent membrane reactor was controlled at a particular methanol to oil molar ratio and catalyst concentration under certain temperature condition. Another proposed model was the work done by Brásio et al. [22] for biodiesel production using mechanical stirring reactor. In view of the economic perspective of biodiesel production, Hassouneh et al. [23] carried out the sensitivity study on the relationships of biodiesel with feedstock price through nonparametric and parametric modeling. Modeling on algae-based feedstock and biocatalyst was conducted as well such as the studies on mass balance of nutrient model [24] and dynamic modeling of immobilized lipase catalyzed biodiesel production [25]. In relation to biodiesel characteristics, Gopinath et al. [26] have established the modeling of biodiesel properties with respect to its iodine value and saponification value.

To date, the kinetics study on methanolysis to produce biodiesel has been focused on irreversible reactions [16, 17]. However, no systematic study on reversible methanolysis is currently available in the literature. This drawback leads to difficulties in the explanation of the intermediate products formed from the methanolysis reaction. To overcome this issue, the aim of this work was to look into the mathematical equations regarding the reversible kinetics in order to produce a valid reversible model of methanolysis reaction. The modeling of the reversible reaction of methanolysis was performed using simulation software, MATLAB, and aimed to evaluate its kinetics model. A statistical analysis of experimental data was also conducted to determine the accuracy of the developed models as compared to that of the experimental data. An optimization routine was then used to adjust the parameters to obtain a good fit to the experimental measurements. The common approach is to minimize a sum of square errors. The multiple range test included in the statistical program was used to prove the existence of homogenous groups within each of the parameters.

## 2. Experimental

### 2.1. Methanolysis

The samples were analyzed to determine the conversion of triglyceride into intermediate and product compounds using gas chromatography (GC) based on a method described in our earlier study [27]. Approximately 0.03 ± 0.005 mL of sample was taken in a 2.0 mL sample vial and diluted with 1.0 mL of ethyl acetate and with 0.5 mL of BSTFA. The vial was then heated in a water bath for 15 min at 40°C. The sample was allowed to cool at room temperature, and it was then injected into the GC system. The capillary column (SGE HT5) 12 m × 0.53 mm and 0.15 *μ*m ID was used in the GC system with hydrogen as carrier gas at a flow rate of 26.7 mL min^−1^ and a split ratio of 1 : 1. The oven temperature was set initially at 80°C and was maintained for 3 min, then was increased at 6°C min^−1^ up to 340°C, and finally maintained at 340°C for another 6 min. The injector and detector temperatures were set at 300°C and 360°C, respectively. The fatty acid composition of JCO is shown in Table 1.

### 2.2. Modeling

To describe the model of biodiesel production using methanolysis process, the mathematical equations were derived in order to produce a valid reversible model of kinetics reaction. Derived mechanisms of JCO were based on methanolysis as shown in the following:

(1) $$\begin{array}{c} \frac{d\left(C_{\text{JTG}}^{}\right)}{dt}=-{k_{1}}_{f}\left(C_{\text{JTG}}\right)\left(C_{\text{MeOH}}\right)+{k_{4}}_{r}\left(C_{\text{JDG}}\right)\left(C_{\text{JME}}\right), \end{array}$$

(2) d(CJDG)dt=+k1f(CJTG)(CMeOH)−k4r(CJDG)(CJME)−k2f(CJDG)(CMeOH)+k5r(CJMG)(CJME),

(3) d(CJMG)dt=+k2f(CJDG)(CMeOH)−k5r(CJMG)(CJME)−k3f(CJMG)(CMeOH)+k6r(CGL)(CJME),

(4) d(CMeOH)dt=−k1f(CJTG)(CMeOH)+k4r(CJDG)(CJME)−k2f(CJDG)(CMeOH)+k5r(CJMG)(CJME)−k3f(CJMG)(CMeOH)+k6r(CGL)(CJME),

(5) d(CJME)dt=+k1f(CJTG)(CMeOH)−k4r(CJDG)(CJME)+k2f(CJDG)(CMeOH)−k5r(CJMG)(CJME)+k3f(CJMG)(CMeOH)−k6r(CGL)(CJME),

(6) $$\begin{array}{c} \frac{d\left(C_{\text{GL}}\right)}{dt}=+k_{3f}\left(C_{\text{JMG}}\right)\left(C_{\text{MeOH}}\right)-{k_{6}}_{r}\left(C_{\text{GL}}\right)\left(C_{\text{JME}}\right), \end{array}$$

where *C* is the concentration of components such as *Jatropha curcas* triglyceride (JTG), *Jatropha curcas* diglyceride (JDG), *Jatropha curcas* monoglyceride (JMG), glycerol (GL), *Jatropha curcas* methyl ester (JME), and methanol (MeOH) and *k* is the effective rate constant of each stepwise reaction.

The resulting kinetics equations, in terms of weight fraction (1)–(6), can be solved by implementing a numerical solution via MATLAB version 7.7. For this study, the ODE solver function, ode45, was selected because it employed nonstiff solutions of the Runge-Kutta method of orders 4 and 5. The rate constants for the three stepwise reactions were determined from the maxima points of the intermediate products and the concentrations of the reaction products at final equilibrium. The reaction products concentrations that fit the product distribution data of concentration versus time for JTG, JDG, JMG, and methyl ester were obtained from the experiment.

### 2.3. Analysis of Variance

The application of an analysis of variance (ANOVA) estimates any statistically significant differences based on the confidence level of 95% (Vega-Gálvez et al., [29]). The accuracy of the models used to correlate the experimental data was evaluated by means of statistical tests such as sum of square error (SSE), root mean square error (RMSE), and Chi-square (*χ* ^2^) as shown in (7). The lowest values of SSE, RMSE, and *χ* ^2^ are selected as optimization criteria in order to evaluate the accuracy of the models,

(7) $$\begin{array}{c} \text{SSE}=\frac{1}{N}\sum_{j=1}^{N}{\left(y_{ej}-y_{mj}\right)}^{2},\\ \text{RMSE}={\left[\frac{1}{N}\sum_{j=1}^{N}{\left(y_{mj}-{y_{e}}_{j}\right)}^{2}\right]}^{1/2},\\ \chi^{2}=\frac{\sum_{j=1}^{N}{\left(y_{ej}-y_{mj}\right)}^{2}}{N-a}, \end{array}$$

where *N*, *y*_*ej*_, *y*_*mj*_, and *a* denote number of data, weight percentage of glycerides by experiment, and weight percentage of glycerides by model and number of constants.

## 3. Results and Discussion

**Figure 2:**
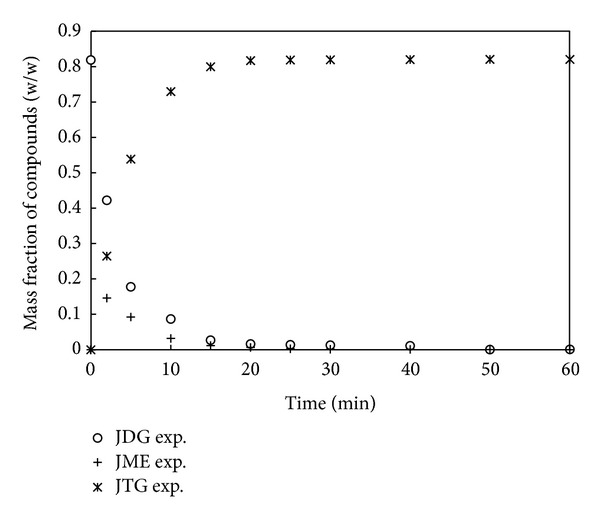
Experimental results from methanolysis process.

The results of analysis by gas chromatography (GC) detected peaks of JTG, JDG, and JME as shown in Figure 2. The simulation curves showed that the formation of methyl esters (biodiesel) was very slow at the beginning and reached the equilibrium value after only 25 min of reaction. As the formation of methyl esters was accompanied by the breakdown of JTG to JDG, there was a sharp drop in concentration of JTG in the first 10 min of reaction. The formation of JME and breakdown of JTG to JDG were observed immediately after 2 min, which indicated that the progressed reaction was very fast. Consequently, the formation and conversion of JDG were also very fast at the beginning, thus reaching a plateau and slowing down as the reaction progresses with time.

**Figure 3:**
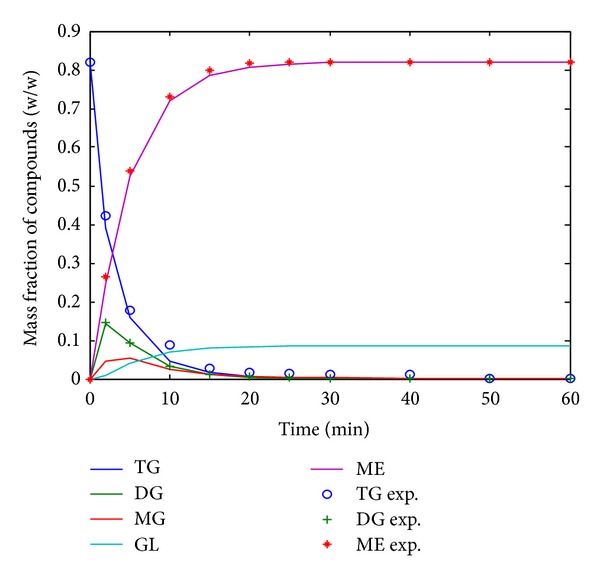
Comparison between experimental and simulation of methanolysis process.

Figure 2 depicted the breakdown of triglyceride slowing down sharply after 10 min of reaction. This phenomenon can be explained later based on the kinetics data. The breakdown of JDG to JMG could not be detected; thus only the modeling result was presented in Figure 3. Likewise, glycerol could not also be detected by GC. After 25 min, the JTG peak disappeared, and the amount of JME stabilized immediately after that.

The experimental weight fractions of each reaction component obtained from the GC analysis were fitted with the kinetic models using the numerical method via Runge-Kutta fourth- and fifth-order method [30]. The rate constants of the proposed kinetics models were determined by minimization of errors based on the optimum criteria of statistical analysis and by comparing the component concentrations at maximum and equilibrium. The simulated concentration profile curves for the kinetics models were then plotted against the experimental values as shown in Figure 3. The dotted points represented experimental data and the solid curves corresponded to the simulated results. Therefore, the kinetic models developed by considering the three reactions individually, as indicated by (1)–(6), would be able to describe the progress of the reaction better than the overall reaction (1). After plotting, all experimental data matched with the simulation results. This fact was supported by the statistical analysis of the reaction.

The irreversible kinetics model published by Yunus and Syam [28] predicted that the *k* values are significantly higher than the *k* values from the present study (Table 2). From Table 2, it can also be seen that the rate constants (*k*) also increased as the reactions progressed through the three stepwise reactions. The rate constants for the forward reactions were considerably higher than those for the reverse reactions. This phenomenon also showed that the rate of first stepwise reaction took place slower than that of the last two reactions. This confirms that the reactions must form an initial complex of compounds before proceeding to form the tetrahedral intermediate with low activation energy consumption. A transition state was required by reactions to break the complex of compounds to be tetrahedral intermediate and the final product. Furthermore, the results also confirmed that the assumption of irreversible reactions for methanolysis of JCO is satisfactory for all conditions.

**Figure 4:**
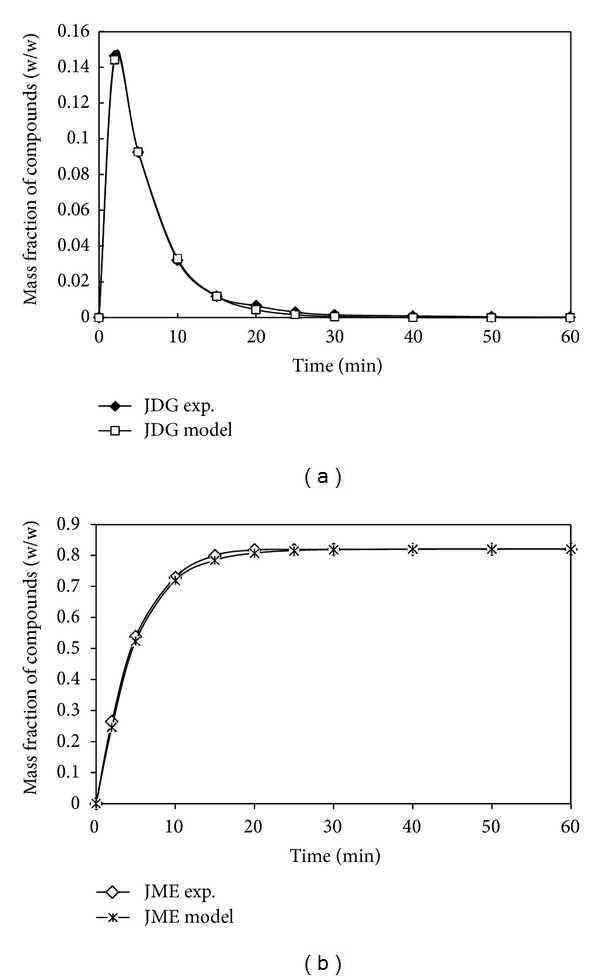
Plot of experimental data and model for (a) JDG and (b) JME compounds.

Figure 4 demonstrated the concentration profiles for diglyceride (JDG) and methyl ester (JME) calculated from the first- order model at 60°C using the *k* values given in Table 2 which was then compared against the experimental values. It was observed that the concentration profile correlated using the current model with MATLAB was fitted well with the experimental data and with the correlation coefficient, *R* ^2^ at 0.99. After optimum curve fitting, the average values of rate constants *k*_1*f*_ and *k*_4*r*_ from the current kinetics models were 7.9 × 10^−2^ and 5.6 × 10^−4^ with levels of uncertainty at 0.0005% and 0.0006%, respectively. On the contrary, the concentration profile for ME was obtained using irreversible model [28], which did not correlate well with the experimental values. Consequently, the resultant correlation coefficient for the integral model was only 0.97, significantly lower than the value obtained from the current kinetic model (0.99). The rate constant *k*_2*f*_ value for the irreversible model was 1.8 × 10^−1^ with a level of uncertainty of 0.01% as compared to 0.150% from the current model. Because the earlier method assumed an irreversible reaction, *k*_2*r*_ is zero whereas *k*_5*r*_ from the current model is 7.5 × 10^−4^. This indicated that the assumption of an irreversible reaction in the integral model is reasonable because the value of *k*_5*r*_ was close to zero.

The average optimum values of *k*_3*f*_ and *k*_6*r*_ were found to be 1.7 × 10^−1^ and 1.4 × 10^−3^ with the levels of uncertainty at 0.0003% and 0.004%, respectively. This confirmed the accuracy of the current kinetics model and also the rate constants determined from this work. However, Table 2 showed the evidence of a reversible reaction, as indicated by the value of *k*_6*r*_ from the current model. The value of *k*_6*r*_ was slightly larger than that of *k*_5*r*_. This confirmed the significance of the reverse reaction. The reverse reaction also led to an accumulation of JDG and a small presence of JMG at the end of the reaction. This problem had been successfully addressed by the current model, which enabled the predication of the JMG concentration profile for the entire 1 h reaction period.

The statistical experimental design has been proposed to allow for adequate variation of operation variables and process responses, allowing for more precise estimation of model parameters and identification of experimental effects. In this context, the use of differential methods in order to avoid the numerical integration of balance equations shall be justified. Most kinetics studies did not take into account the variance of the measured values, and in this matter, statistical tests cannot be applied properly to evaluate the model adequacy and the quality of model parameters. Additional evidence supported that the validity of the current kinetics models was the statistical evaluation of the models based on curve fitting as depicted in Table 3. The model exhibited good statistical correlation with a sum of squares error (SSE) of 4.3 × 10^−6^, RMSE of 2.1 × 10^−3^, and Chi-square (*χ*²) of 5.3 × 10^−6^, compared to the experimental JDG values. The correlation coefficient *R* ^2^ was relatively higher at 0.98. The results indicated that the mean values of statistical analyses applied to the kinetic models for the reversible reactions expressed a good fit with values close to zero for all statistical tests as shown in Table 3. In general, the statistical analysis indicated that the current model improved the estimation of the concentration profiles of components for the methanolysis process.

**Figure 5:**
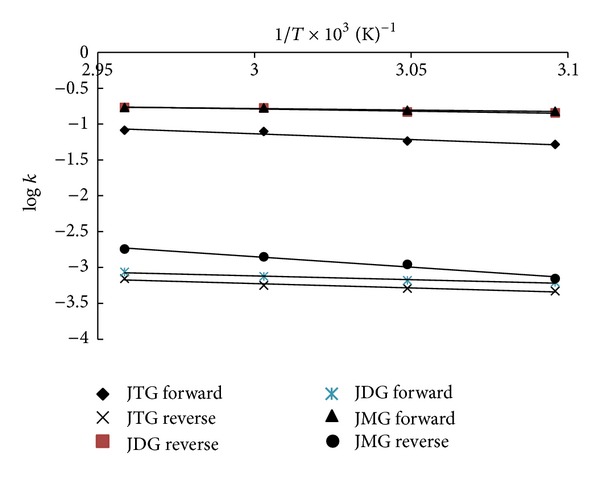
Arrhenius activation energy at various temperatures and rate constants.

Figure 5 showed the plot of Arrhenius activation energy under several reaction rate constants and reaction temperatures. The values of activation energy for the reversible reaction of methanolysis were found to be within the range of 6.5–44.4 KJ mol^−1^ as presented in Table 4. The activation energies of forward reactions are generally lower than those of the reverse reactions, thus the forward reactions are more favored. Based on the underlying principle of activation energy, the mechanism of reversible reaction can be understood in greater detail. For the forward reaction between JCO and methanol, the reaction should acquire the activation energy level before the reactants can form the activated complex.

Similarly, for the reverse reaction, glycerol and methyl ester must also acquire the activation energies level prior to forming the activated complex. These trends showed that the forward reactions required less energy to form the complex as compared to the reverse reactions which need more energy to form the activated complex. As a result, the rate of forward reactions proceeds faster than the rate of reverse reactions due to the lower level of activation energy.

## 4. Conclusion

In this study, the dynamic modeling of reversible methanolysis reaction for JCO using MATLAB was successfully performed. The kinetics models incorporated reversible reactions, which enable better prediction of reaction rate parameters. The results indicated that the homogenous alkaline-catalyzed methanolysis reaction took place relatively fast. The forward reactions occurred at a much faster rate than the reverse reactions. The rate of breakdown of JTG into JDG was slower than the breakdown of JDG into JMG and finally the formation of methyl ester. These were evidenced from the values of the rate constants for the individual stepwise reactions. The activation energies for the reverse reactions were generally higher than those for the forward reactions which indicated that the reverse reactions were more difficult to take place than the forward reaction. In addition, the rates of reverse reaction were also significantly slower than those of the forward reaction. The statistical analysis demonstrated that the models correlated well with the experimental data. The values of statistical parameters were very close to zero which demonstrated that the kinetics models developed using MATLAB and its corresponding parameters were reliable and accurate.

## Figures and Tables

**Table 1:** Fatty acid composition of JCO.

Fatty acids	Formula	C : D	(wt%)
Myristic	C_14_H_28_O_2_	14 : 0	0.1
Palmitic	C_16_H_32_O_2_	16 : 0	13.9
Palmitoleic	C_16_H_30_O_2_	16 : 1	0.8
Margaric	C_17_H_34_O_2_	17 : 0	0.1
Stearic	C_18_H_36_O_2_	18 : 0	7.7
Oleic	C_18_H_34_O_2_	18 : 1	48.0
Linoleic	C_18_H_32_O_2_	18 : 2	28.7
Linolenic	C_18_H_30_O_2_	18 : 3	0.1
Arachidic	C_20_H_40_O_2_	20 : 0	0.3
Gadoleic	C_20_H_38_O_2_	20 : 1	0.1
Behenic	C_22_H_44_O_2_	22 : 0	0.2

C: carbon number; Y: double bonds number.

**Table 2:** Rate constant *k* for reversible reaction model at 60°C.

Rate constant, min^−1^	Values	Yunus and Syam (2011) [28]
*k*_1*f*_	7.9 × 10^−2^	1.5 × 10^−1^
*k*_2*f*_	1.5 × 10^−1^	1.8 × 10^−1^
*k*_3*f*_	1.7 × 10^−1^	—
*k*_4*r*_	5.6 × 10^−4^	—
*k*_5*r*_	7.5 × 10^−4^	—
*k*_6*r*_	1.4 × 10^−3^	—

**Table 3:** Values of statistical parameters for JDG and JME.

Parameters	JDG	JME
SSE	4.3 × 10^−6^	4.7 × 10^−4^
RMSE	2.1 × 10^−3^	2.2 × 10^−2^
*χ* ^2^	5.3 × 10^−6^	5.2 × 10^−5^

**Table 4:** Activation energies for reversible reaction of methanolysis.

Scenario of reaction	Rate constants	Activation energies (KJ mol^−1^)	*r* ^2^
JTG + MeOH → JDG + JME	*k*_1*f*_	6.5	0.91
MeOH + JTG ⇠ JME + JDG	*k*_4*r*_	39.5	0.92
JDG + MeOH → JMG + JME	*k*_2*f*_	6.1	0.92
MeOH + JDG ⇠ JME + JMG	*k*_5*r*_	24	0.97
JMG + MeOH → GL + JME	*k*_3*f*_	2.4	0.92
MeOH + JMG ⇠ JME + GL	*k*_6*r*_	44.4	0.97

## References

[1] Syam AM, Yunus R, Ghazi TIM, Yaw TCS (2009). Methanolysis of jatropha oil in the presence of potassium hydroxide catalyst. *Journal of Applied Sciences*.

[2] Dias JM, Alvim-Ferraz MCM, Almeida MF (2008). Comparison of the performance of different homogeneous alkali catalysts during transesterification of waste and virgin oils and evaluation of biodiesel quality. *Fuel*.

[3] Syam AM, Yunus R, Ghazi TIM, Choong TSY (2012). Synthesis of *Jatropha curcas* oil-based biodiesel in a pulsed loop reactor. *Industrial Crops and Products*.

[4] Lotero E, Liu Y, Lopez DE, Suwannakarn K, Bruce DA, Goodwin JG (2005). Synthesis of biodiesel via acid catalysis. *Industrial and Engineering Chemistry Research*.

[5] Marchetti JM, Miguel VU, Errazu AF (2007). Possible methods for biodiesel production. *Renewable and Sustainable Energy Reviews*.

[6] Sonare NR, Rathod VK (2010). Transesterification of used sunflower oil using immobilized enzyme. *Journal of Molecular Catalysis B*.

[7] Liu Y, Xin H-L, Yan Y-J (2009). Physicochemical properties of stillingia oil: feasibility for biodiesel production by enzyme transesterification. *Industrial Crops and Products*.

[8] Ilgen O (2011). Dolomite as a heterogeneous catalyst for transesterification of canola oil. *Fuel Processing Technology*.

[9] Li E, Xu ZP, Rudolph V (2009). MgCoAl-LDH derived heterogeneous catalysts for the ethanol transesterification of canola oil to biodiesel. *Applied Catalysis B*.

[10] Taufiq-Yap YH, Lee HV, Yunus R, Juan JC (2011). Transesterification of non-edible *Jatropha curcas* oil to biodiesel using binary Ca-Mg mixed oxide catalyst: effect of stoichiometric composition. *Chemical Engineering Journal*.

[11] Borges ME, Díaz L (2012). Recent developments on heterogeneous catalysts for biodiesel production by oil esterification and transesterification reactions: a review. *Renewable and Sustainable Energy Reviews*.

[12] Helwani Z, Othman MR, Aziz N, Kim J, Fernando WJN (2009). Solid heterogeneous catalysts for transesterification of triglycerides with methanol: a review. *Applied Catalysis A*.

[13] Cheng L-H, Yen S-Y, Chen Z-S, Chen J (2012). Modeling and simulation of biodiesel production using a membrane reactor integrated with a prereactor. *Chemical Engineering Science*.

[14] Darnoko D, Cheryan M (2000). Kinetics of palm oil transesterification in a batch reactor. *Journal of the American Oil Chemists’ Society*.

[15] Vicente G, Martínez M, Aracil J (2006). Kinetics of *Brassica carinata* oil methanolysis. *Energy and Fuels*.

[16] Stamenković OS, Todorović ZB, Lazić ML, Veljković VB, Skala DU (2008). Kinetics of sunflower oil methanolysis at low temperatures. *Bioresource Technology*.

[17] Berchmans HJ, Morishita K, Takarada T (2010). Kinetic study of methanolysis of *Jatropha curcas*-waste food oil mixture. *Journal of Chemical Engineering of Japan*.

[18] Noureddini H, Zhu D (1997). Kinetics of transesterification of soybean oil. *Journal of the American Oil Chemists’ Society*.

[19] Vicente G, Martínez M, Aracil J, Esteban A (2005). Kinetics of sunflower oil methanolysis. *Industrial and Engineering Chemistry Research*.

[20] Chen H, Wang J (2005). Kinetics of KOH catalyzed trans-esterification of cottonseed oil for biodiesel production. *Journal of Chemical Industry and Engineering*.

[21] Abeynaike A, Sederman AJ, Khan Y, Johns ML, Davidson JF, Mackley MR (2012). The experimental measurement and modeling of sedimentation and creaming for glycerol/biodiesel droplet dispersions. *Chemical Engineering Science*.

[22] Brásio ASR, Romanenko A, Santos LO, Fernandes NCP (2011). Modeling the effect of mixing in biodiesel production. *Bioresource Technology*.

[23] Hassouneh I, Serra T, Goodwin BK, Gil JM (2012). Non-parametric and parametric modeling of biodiesel, sunflower oil, and crude oil price relationships. *Energy Economics*.

[24] Rösch C, Skarka J, Wegerer N (2012). Materials flow modeling of nutrient recycling in biodiesel production from microalgae. *Bioresource Technology*.

[25] Al-Zuhair S, Dowaidar A, Kamal H (2009). Dynamic modeling of biodiesel production from simulated waste cooking oil using immobilized lipase. *Biochemical Engineering Journal*.

[26] Gopinath A, Puhan S, Nagarajan G (2009). Theoretical modeling of iodine value and saponification value of biodiesel fuels from their fatty acid composition. *Renewable Energy*.

[27] Yunus R, Lye OT, Fakhru’l-Razi A, Basri S (2002). A simple capillary column GC method for analysis of palm oil-based polyol esters. *Journal of the American Oil Chemists’ Society*.

[28] Yunus R, Syam AM (2011). Kinetics of the transesterification of *Jatropha curcas* triglyceride with an alcohol in the presence of an alkaline catalyst. *International Journal of Sustainable Energy*.

[29] Vega-Gálvez A, Notte-Cuello E, Lemus-Mondaca R, Zura L, Miranda M (2009). Mathematical modelling of mass transfer during rehydration process of Aloe vera (*Aloe barbadensis* Miller). *Food and Bioproducts Processing*.

[30] Abd Hamid H, Yunus R, Choong TSY (2010). Utilization of MATLAB to simulate kinetics of transesterification of palm oil-based methyl esters with trimethylolpropane for biodegradable synthetic lubricant synthesis. *Chemical Product and Process Modeling*.

